# Role of data measurement characteristics in the accurate detection of Parkinson’s disease symptoms using wearable sensors

**DOI:** 10.1186/s12984-020-00684-4

**Published:** 2020-04-20

**Authors:** Nicholas Shawen, Megan K. O’Brien, Sanjeev Venkatesan, Luca Lonini, Tanya Simuni, Jamie L. Hamilton, Roozbeh Ghaffari, John A. Rogers, Arun Jayaraman

**Affiliations:** 1 Max Nader Lab for Rehabilitation Technologies and Outcomes, Shirley Ryan AbilityLab, Chicago, IL 60611 USA; 2 Feinberg School of Medicine, Northwestern University, Chicago, IL 60611 USA; 3 Department of Physical Medicine and Rehabilitation, Northwestern University, Chicago, IL 60611 USA; 4 Department of Computer Science, University of Illinois at Urbana-Champagne, Urbana, IL 61801 USA; 5 Department of Neurology, Northwestern University, Chicago, IL 60611 USA; 6 The Michael J. Fox Foundation for Parkinson’s Research, New York, NY 10120 USA; 7 Center for Bio-Integrated Electronics, Departments of Materials Science and Engineering, Biomedical Engineering, Chemistry, Mechanical Engineering, Electrical Engineering and Computer Science, Neurological Surgery, Simpson Querrey Institute for Nano/Biotechnology, McCormick School of Engineering, Feinberg School of Medicine, Northwestern University, Evanston, IL 60208 USA; 8 Department of Physical Therapy and Human Movement Sciences, Northwestern University, Chicago, IL 60611 USA

**Keywords:** Parkinson’s disease, Wearable sensors, Soft wearables, Machine learning, Symptom detection, Tremor, Bradykinesia, Daily activities

## Abstract

**Background:**

Parkinson’s disease (PD) is a progressive neurological disease, with characteristic motor symptoms such as tremor and bradykinesia. There is a growing interest to continuously monitor these and other symptoms through body-worn sensor technology. However, limited battery life and memory capacity hinder the potential for continuous, long-term monitoring with these devices. There is little information available on the relative value of adding sensors, increasing sampling rate, or computing complex signal features, all of which may improve accuracy of symptom detection at the expense of computational resources. Here we build on a previous study to investigate the relationship between data measurement characteristics and accuracy when using wearable sensor data to classify tremor and bradykinesia in patients with PD.

**Methods:**

Thirteen individuals with PD wore a flexible, skin-mounted sensor (collecting tri-axial accelerometer and gyroscope data) and a commercial smart watch (collecting tri-axial accelerometer data) on their predominantly affected hand. The participants performed a series of standardized motor tasks, during which a clinician scored the severity of tremor and bradykinesia in that limb. Machine learning models were trained on scored data to classify tremor and bradykinesia. Model performance was compared when using different types of sensors (accelerometer and/or gyroscope), different data sampling rates (up to 62.5 Hz), and different categories of pre-engineered features (up to 148 features). Performance was also compared between the flexible sensor and smart watch for each analysis.

**Results:**

First, there was no effect of device type for classifying tremor symptoms (*p* > 0.34), but bradykinesia models incorporating gyroscope data performed slightly better (up to 0.05 AUROC) than other models (*p* = 0.01). Second, model performance decreased with sampling frequency (*p* < 0.001) for tremor, but not bradykinesia (*p* > 0.47). Finally, model performance for both symptoms was maintained after substantially reducing the feature set.

**Conclusions:**

Our findings demonstrate the ability to simplify measurement characteristics from body-worn sensors while maintaining performance in PD symptom detection. Understanding the trade-off between model performance and data resolution is crucial to design efficient, accurate wearable sensing systems. This approach may improve the feasibility of long-term, continuous, and real-time monitoring of PD symptoms by reducing computational burden on wearable devices.

## Background

Parkinson’s disease (PD) is a neurodegenerative disorder characterized by progressive motor symptoms such as tremors, rigidity, and bradykinesia (slowness of movement). It is estimated that the number of people affected by PD worldwide has more than doubled from 1990 to 2016, making it the fastest growing neurological disease [1, 2]. Available treatments for PD motor deficits continue to expand, including pharmacological, surgical, and other therapeutic interventions [3]. As the disease progresses, changes to an individual’s customized treatment plan are often needed to maintain symptom control as medications wear off while avoiding troublesome side-effects. Long duration, continuous monitoring of PD symptoms would allow for more personalized treatment and better control of symptoms during the time-course of the disease.

Motor symptoms of PD are typically assessed via periodic, in-person evaluations by a clinician, with supplemental diaries completed by patients [4, 5]. However, evaluations are infrequent and sometimes inaccurate [6], and the diaries can be cumbersome and are often not maintained over long periods of time [7, 8]. Pilot investigations have explored the use of telemedicine to replace or accompany in-person evaluations, though the frequency of assessment would still be limited by patient/clinician schedules [9–11].

Wearable devices offer a powerful alternative to traditional, in-person clinical assessment strategies. These devices can house multiple types of sensors to continuously record physiological data related to PD symptoms. Supervised machine learning models can be trained on this data to detect the presence and severity of a symptom offline or in real time. There are many potential algorithms to associate features of the data signals with a diagnostic output, which is typically a problem of classifying the data (e.g., as having the presence/absence of a symptom, or whether the symptom is mild/moderate/severe). Once trained, these models are used to detect symptom presence and severity for new data. Modeling motor symptoms of PD is primarily conducted using sensors that record body movements, such as accelerometers, gyroscopes, or electromagnetic motion trackers [12–16]. Other types of sensors, including those measuring bioelectrical activity (electromyography, electroencephalography), have also been used [17–20]. Results from current state-of-the-art models are encouraging, with accuracies exceeding 85% for detection of tremor and bradykinesia during controlled tasks or free movements [16, 21]. These recent results indicate that wearable technologies have become increasingly viable for monitoring PD symptoms in the clinic and community.

However, the real-world implementation and utility of these systems is heavily limited by practical considerations, including the battery life and memory capacity of the devices. The need to remove and replace devices – to restore the battery or download collected data, for example – can lead to intermittent usage or inconsistent device positioning. Accelerometers are typically considered the standard or minimum necessary sensor configuration for characterizing human activity. These sensors measure movement data while consuming relatively little power compared to other types of inertial sensors, like gyroscopes, and can meaningfully describe human movements at sampling rates much lower than those used for bioelectric recordings. It is unclear whether accelerometer data alone is sufficient to detect PD symptoms, or whether additional sensor types are necessary to characterize a symptom and improve detection. In addition, sampling rates of wearable sensors can be tuned, increasing temporal resolution and thereby model accuracy at the cost of some increase in power and memory overhead. Finally, features of the data signals may be computed onboard the device and stored in place of raw signals to conserve memory or provide real-time symptom tracking; however, the feasibility of real-time tracking is predicated on the complexity of the features themselves and available system resources. Features based on relatively complex signal processing techniques, such as fast Fourier transforms or sample entropy, may capture subtly meaningful characteristics of a signal but also require more time to compute and drain device power more quickly. Together, these ideas suggest an important tradeoff between the relative costs (i.e. power consumption, device memory) and benefits (i.e. accuracy of symptom detection) of data complexity in disease monitoring.

This study evaluates the impact of data measurement characteristics on the accuracy of PD symptom detection. Individuals with PD wore a flexible, skin-mounted sensor that recorded accelerometer and gyroscope data from the hand during various motor tasks. Machine learning models were used to classify the presence and severity of tremor and bradykinesia symptoms based on scores from an experienced, licensed clinician. We characterized changes in model performance for different types of sensors, sampling rates, and subsets of a pre-engineered feature set. Performance was compared between the flexible sensor on the hand and a consumer-grade smart watch recording accelerometer data only. Although not an exhaustive exploration of data measurement characteristics for wearable devices, our results show that simplified data collection approaches can be used for PD symptom monitoring without compromising accuracy.

## Methods

This work is a continuation of the “Wireless Adhesive Sensor Sub-Study,” which is part of a larger multi-center study entitled “Clinician Input Study on Parkinson’s Disease” (CIS-PD), sponsored by the Michael J. Fox Foundation for Parkinson’s Research.

### Participants

Twenty individuals diagnosed with PD participated in the study. The study was approved by the Institutional Review Board of Northwestern University (Chicago, IL; IRB No. STU00203796), and all participants provided written informed consent. Analysis for this study was limited to 13 participants who simultaneously wore the flexible sensor and a smart watch during assessment. Demographic and clinical characteristics of these study participants are summarized in Table 1.

**Table 1:** Participant demographics and clinical summary

Participant Characteristics	Values
Sex (female/male)	4 / 9
Age (years)	62.1 ± 10.7
Time since diagnosis (years)	6.4 ± 4.5
Fluctuator (yes/no)	5 / 8
Side predominantly affected at first assessment (right/left/bilateral)	2 / 8 / 3
MDS part III score, day 1 OFF medication	28.8 ± 10.2
MDS part III score, day 1 ON medication	17.9 ± 6.8
MDS part III score, day 2 ON medication	19.6 ± 6.0

Relevant demographic characteristics of study participants included in this analysis. Values are presented as Mean ± Standard Deviation where applicable. Total participants (N) was 13.

### Study protocol

**Fig. 1:**
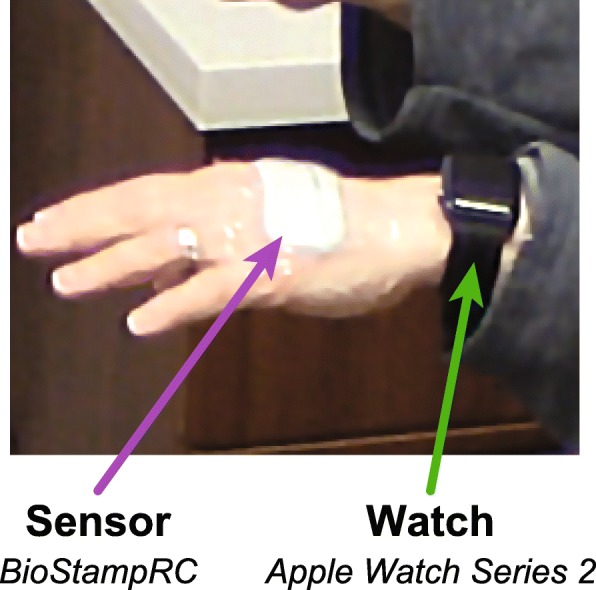
Wearable device placement. Participants wore a flexible BioStampRC sensor recording accelerometer and gyroscope data on the dorsal aspect of the hand, secured with adhesive dressing. They also wore an Apple Watch recording accelerometer data on the wrist

Participants wore a BioStampRC sensor (MC10 Inc., Lexington, MA, USA) – a lightweight, flexible, sensor that can support multiple sensing modalities – on the dorsal aspect of the hand on their predominantly affected side (Fig. 1). The sensor was secured to the skin with adhesive dressing. They also wore a smart watch (Apple Watch Series 2, Apple Inc., Cupertino, CA) on the same-side wrist during all assessments. The predominantly affected side was determined by clinician assessment using the Movement Disorder Society – Unified Parkinson’s Disease Rating Scale (MDS-UPDRS). Participants who were bilaterally affected wore the devices on their dominant side. Participants wore the devices for up to 3 h without need to replace the devices. The BioStampRC recorded tri-axial data from an accelerometer (range: ±4G) and gyroscope (range: ±1000°/s) at a sampling rate of 62.5 Hz. The smart watch recorded tri-axial accelerometer data using the ResearchKit framework at an average rate of 50 Hz.

These participants also wore additional BioStampRC sensors as part of a larger study, with sensors placed bilaterally on the arm, hand, and thigh [22]. For simplicity, we focus all analyses here on the data from the single hand-worn BioStampRC and smart watch on the predominantly affected side.

PD symptoms were assessed during 13 different standardized motor tasks, which included functional tasks (e.g., walking for 60 s), fine upper extremity tasks (e.g., typing), gross upper extremity tasks (e.g., pouring water), and tasks used in clinical assessment (e.g., finger to nose). Participants were given only general instructions for each task to encourage them to perform each task as naturally as possible. Additional details about these activities are available in [22]. A trained clinician rated the severity of tremor and bradykinesia in each arm during the tasks, based on a 0–4 scale using score descriptions based on the MDS-UPDRS Part III. Clinician ratings from the predominantly affected side (or, for bilaterally affected, the dominant side) were considered to be the ground truth label for machine learning models.

Each participant completed seven repetitions of each task across two clinical visits. During the first visit, participants arrived in the OFF medication state (approximately 12 h after last dose of medication) in order to maximize motor symptoms. After performing each task once, participants then took one dose of their medication and repeated each task five more times at 30-min intervals as the medication took effect (ON medication state). The second visit occurred about 2 weeks later at the same time of day as the first visit; participants arrived in the ON medication state, taking their medication as usual, and performed each task once. Data from both visits were included for analysis to capture a broad range of symptom presentations.

### Feature extraction

To correct for occasional dropped samples and non-uniform sampling rates, data recordings from each task were interpolated to the intended sampling rate (62.5 Hz for BioStampRC, 50 Hz for smart watch) using a cubic spline interpolation. For analyses using data at reduced sampling rates, these signals were then down-sampled using a polyphase filtering approach. Accelerometer and gyroscope recordings were then segmented into 5-s clips with 50% overlap. This was done to standardize the individual clips and provide several clips from each task recording, which ranged in length from about 15 s to 60 s. Clips with less than 80% of the expected number of samples in the un-interpolated data were discarded. This process yielded 16,445 and 14,339 clips from the sensor and smart watch datasets, respectively (Table 2). To remove effect of hand orientation, accelerometer data were also high-pass filtered with a cutoff at 0.5 Hz.

**Table 2:** Number of data clips scored for tremor and bradykinesia used in the supervised machine learning models

Score	No. Clips with Tremor Score (%)		No. Clips with Bradykinesia Score (%)	
	*BioStampRC*	*Watch*	*BioStampRC*	*Watch*
0	12,143 (73.8%)	10,485 (73.1%)	5487 (45.0%)	4845 (44.8%)
> 0	4302 (26.2%)	3854 (26.9%)	6697 (55.0%)	5979 (55.2%)
1	2684 (16.3%)	2346 (16.4%)	4764 (39.1%)	4213 (38.9%)
2	1274 (7.7%)	1233 (8.6%)	1835 (15.1%)	1676 (15.5%)
3	344 (2.0%)	275 (1.9%)	98 (0.8%)	90 (0.8%)
4	0 (0.0%)	0 (0.0%)	0 (0.0%)	0 (0.0%)
***Total***	***16,445***	***14,339***	***12,184***	***10,824***

Number of 5-s clips for tremor and bradykinesia symptoms, by score and device type. Fewer clips are available with bradykinesia scores because not all tasks involved enough movement for the clinician to assess bradykinesia

We computed features for each data clip, which included separate features on the three axes of the accelerometer and gyroscope signals, as well as on the magnitude of the signals. This resulted in 74 features per sensor type per clip, which we categorized into the 5 feature categories given in Table 3. These features were chosen as an expansion of features we had used in a prior analysis [22].

**Table 3:** Feature categorization for supervised machine learning models

Feature category	Abbreviation	Features	No. Tri-axial features	No. Magnitude features
Time	T	Root mean square, range, mean, variance, skew, kurtosis	18	6
Frequency	F	Dominant frequency, Relative magnitude, Moments of power spectral density (mean, standard deviation, skew, kurtosis)	18	6
Entropy	E	Sample entropy	3	1
Correlation	C	Cross-correlation peak (XY,XZ,YZ), Cross-correlation lag (XY,XZ,YZ)	6	0
Derivative	D	Moments of the signal derivative (mean, standard deviation, skew, kurtosis)	12	4
***Total for each sensor type***			***57***	***17***

Features extracted from both accelerometer and gyroscope data signals and used as inputs for symptom models. Features are shown split into the categories used during the analysis of feature types

### Classification models

We used random forest (RF) machine learning models to classify PD symptoms. RF models are advantageous due to their high performance, low number of hyperparameters, and ability to reduce overfitting. We have also previously explored convolutional neural networks for symptom detection, but did not see any substantial improvement relative to the RF models [22]. The number of trees in each RF model was set to 50, based on a prior analysis of out-of-bag training error using another subset of data from this study [22].

Models were built using a population-based, leave-one-participant-out (LOPO) approach – that is, applying training data from all participants but one to classify tremor and bradykinesia in the left-out participant. Cross-validation across all possible LOPO folds was used to estimate the distribution of performance metrics. Model performance was evaluated by Area Under the Receiver Operator Characteristic curve (AUROC), where values closer to 1.0 indicate the model is better able to distinguish the presence or absence of the symptom classification, and values closer to 0.5 indicate performance closer to chance.

We examined two types of RF models (binary and multiclass) for each PD symptom examined (tremor and bradykinesia), resulting in four models total:

- A binary model classifies the presence or absence of the PD symptom. A symptom was determined to be present if the clinician gave a 1–4 rating in the MDS-UPDRS, and considered absent if the clinician gave a 0 rating. Model performance was determined by the AUROCs computed from each participant’s data.
- A multiclass model scores the PD symptom on the same 0–4 scale used by the clinician based on the MDS-UPDRS. Model performance was determined by the weighted average of AUROCs computed on each of the classes, 0–4, for each participant’s data.

### Comparison of sensor types

To evaluate the relative contribution of data from the accelerometer and gyroscope sensors of the BioStampRC, we compared AUROCs using the 74 features derived from either accelerometer (Accel) or gyroscope (Gyro) data alone, or using the 148 features combined from both sensor types (Combo). We also examined a model using the 74 accelerometer features from the smart watch (Watch). Comparisons were made for both binary and multiclass models trained to classify either tremor or bradykinesia. For each of these model categories, a one-way repeated measures analysis of variance (rmANOVA) tested for significant effect of sensor type on model performance. The significance level *α* was set to 0.05 for this test. If a significant effect was found, paired t-tests were applied to make the following post-hoc pairwise comparisons: Combo vs. Accel, Combo vs. Gyro, Combo vs. Watch, and Accel vs. Watch. One-tailed tests were applied to assess whether the Combo condition provided any significant benefit over the other conditions. A two-tailed test was applied to compare Accel vs. Watch, testing for significant differences in performance resulting from accelerometer hardware (BioStampRC vs. Apple smart watch) or location (hand vs. wrist). For each set of comparisons for a single model type/symptom combination, the Holm-Bonferroni correction was used to control the family-wise error rate at *α* =0.05.

### Comparison of sampling rates

To evaluate the effect of reduced sampling rate, we interpolated signals from the BioStampRC and smart watch to eight lower frequencies: 50 (skin-adhesive sensor only), 40, 30, 20, 10, 7.5, and 5 Hz. As with the preceding analysis, a one-way rmANOVA was used to assess for significant effect of resampled rate on the performance of symptom models (*α* =0.05). If a significant effect was observed, one-tailed paired t-tests were used to make pairwise comparisons to assess significant decrease in performance from the original sampling rate to all lower frequencies. The Holm-Bonferroni correction was again applied to control the family-wise error rate (*α* =0.05).

### Comparison of feature categories

The trade-off between the cost of computing more features and the potential increase in model performance enabled by these features, becomes particularly salient for online computations and real-time monitoring. To examine this relationship, we trained models using all possible combinations of the feature groups listed in Table 3. As a proxy for computational cost, we used the average computation time for each combination of features. Features were computed in Python 3.6 using NumPy and SciPy libraries on a desktop computer running Windows 10. These values will not necessarily translate to other contexts, particularly embedded systems, but can be illustrative for a general examination of the relationship between feature complexity and performance. We chose to perform selection on feature categories rather than individual features for two main reasons: (1) individual ranking of features would potentially give spurious results for some features that do not generalize well beyond our data, and (2) for some feature categories, such as frequency-domain measures, a portion of the computational cost stems from a shared preliminary computation, such as a Fourier transform. Thus, it may be more practical to discuss relative contributions of feature categories with shared or similar computations.

Any feature set that did not have higher average AUROC than all sets with lower computation time was discarded to identify the subset of feature combinations with only increasing performance for greater computation time. We used one-way rmANOVA to assess for significant effect of feature combination within each model category (*α* =0.05). If significant, one-tailed paired t-tests with Holm-Bonferroni correction were used to compare performance of the feature set with highest computation time to the performances of each other available feature set (family-wise *α* =0.05).

### Comparison of tri-axial and magnitude-based features

Computational costs could also be reduced by using features based only on signal magnitude, rather than on tri-axial signals. We repeated the above analysis using only the magnitude-based features from each group, except the cross-correlation features were kept as before since no magnitude-based features were available for this feature category. The peak performance when using magnitude features only was compared to performance when using both magnitude and tri-axial features using a paired t-test for each model category.

### Code availability

All data processing and analysis for this study was done using custom code run using the Anaconda distribution of Python 3.6. The code used along with example data is available as a GitHub repository (https://github.com/nshawen/DataCharacteristics_PD).

## Results

### Sensor type analysis

**Fig. 2:**
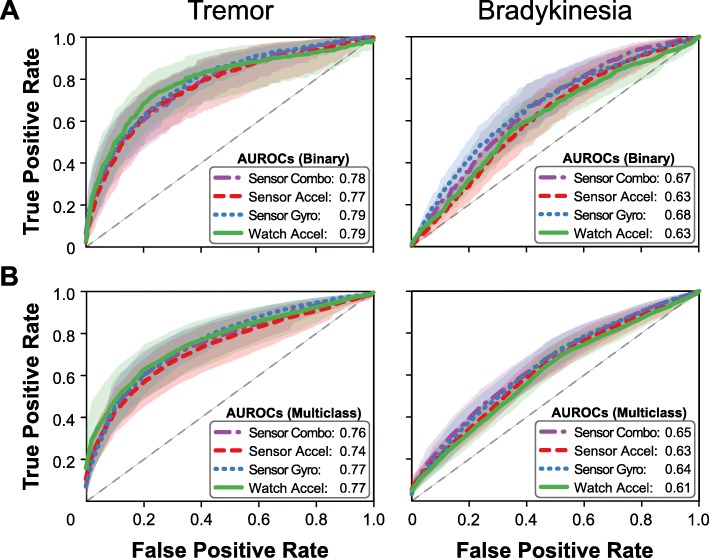
Effect of sensor set. ROC curves for (**a**) Binary and (**b**) Multiclass models of tremor and bradykinesia with average AUROC. Accelerometer data is sufficient to classify tremor, whereas the combination of gyroscope and accelerometer data improves detection of bradykinesia

We first examined differences in model performance when trained using data obtained from different sets of sensors. Receiver operating characteristic (ROC) curves for each model are shown in Fig. 2, and AUROC for each sensor group is detailed in Table 4. Models estimating tremor symptoms showed no significant differences in performance across data sources for either Binary or Multiclass models (rmANOVA: Binary: F = 0.57, *p* = 0.63; Multiclass: F = 1.17, *p* = 0.34). The range of AUROC values across these models was also small (Binary: 0.02, Multiclass: 0.03), and there was no apparent trend between sensor type and performance. For Binary models of bradykinesia symptoms, models using data from the gyroscope sensor (Combo, Gyro) performed significantly better than models using only Watch or accelerometer data (rmANOVA: F = 4.98, *p* = 0.01; paired t-tests: Combo vs. Watch: *p* = 0.001, Combo vs. Accel: *p* = 0.007). The effect of sensor type on AUROC was also somewhat larger in magnitude for this symptom (range: 0.05). There were no significant differences in performance between combined sensors and gyroscope alone (*p* = 0.38) or sensor accelerometer and smart watch (*p* = 0.39). No significant differences due to sensor type were observed for Multiclass models (rmANOVA, F = 2.09, *p* = 0.12), although models including gyroscope data still performed better than models using only accelerometer data (ΔAUROC: 0.01–0.04).

**Table 4:** Effect of sensor set on model performance

Sensor Set	Tremor		Bradykinesia	
	*Binary*	*Multiclass*	*Binary*	*Multiclass*
Combo	0.78 (0.70–0.86)	0.76 (0.68–0.83)	0.67 (0.61–0.74)	0.65 (0.59–0.71)
Accel	0.77 (0.67–0.87)	0.74 (0.65–0.82)	0.63 (0.57–0.70)	0.63 (0.57–0.68)
Gyro	0.79 (0.74–0.85)	0.77 (0.72–0.82)	0.68 (0.61–0.75)	0.64 (0.59–0.70)
Watch	0.79 (0.69–0.89)	0.77 (0.68–0.86)	0.63 (0.56–0.69)	0.61 (0.56–0.66)

Average and 95% confidence intervals of model performance (AUROC) to classify PD symptoms using different sensor sets

From this analysis, we found that pairing gyroscope and accelerometer data improved detection of bradykinesia (Combo), whereas accelerometer data alone was sufficient for detecting tremor (Accel and Watch). For simplicity, the subsequent analyses were performed using only the sensor set identified here, as well as the smart watch data as a continued comparison.

### Sampling rate analysis

Using the sensor types determined from the sensor type analysis, we next sought to assess the minimum necessary sampling rate required to detect each PD symptom. We simulated lower sampling rate by downsampling the data to a target rate, then calculated features for training and testing the RF models. One-way rmANOVA and paired t-tests were then applied to determine at which sampling frequencies model performance differed significantly from AUROC at the original (maximum) sampling rate (Table 5).

**Table 5:** Effect of sampling rate on model performance

Sampling Rate (Hz)	Tremor		Bradykinesia	
	*Binary*	*Multiclass*	*Binary*	*Multiclass*
62.5	0.77 (0.67, 0.87)	0.74 (0.65, 0.82)	0.67 (0.61, 0.74)	0.65 (0.59, 0.71)
50	0.77 (0.67, 0.87)	0.74 (0.66, 0.83)	0.68 (0.61, 0.74)	0.65 (0.59, 0.70)
40	0.77 (0.67, 0.87)	0.75 (0.66, 0.84)	0.68 (0.61, 0.74)	0.65 (0.59, 0.70)
30	**0.76 (0.66, 0.86)**	**0.74 (0.66, 0.83)**	**0.68 (0.61, 0.74)**	**0.65 (0.59, 0.70)**
20	0.75 (0.65, 0.85)*	0.73 (0.64, 0.81)	0.67 (0.61, 0.74)	0.65 (0.59, 0.70)
10	0.73 (0.64, 0.82)*	0.70 (0.62, 0.78)*	0.68 (0.61, 0.74)	0.64 (0.58, 0.70)
7.5	0.72 (0.63, 0.81)*	0.69 (0.61, 0.77)*	0.69 (0.62, 0.75)	0.65 (0.60, 0.70)
5	0.70 (0.62, 0.79)*	0.70 (0.62, 0.78)*	0.67 (0.60, 0.74)	0.65 (0.60, 0.71)

Average and 95% confidence intervals of model performance (AUROC) to classify PD symptoms using different sampling rates for the Accel (tremor) or Combo (bradykinesia) sensor types. Asterisk (*) indicates significant difference from performance at the original sampling rate. Bolded results indicate the sampling rate selected for subsequent analyses

All models of tremor symptoms showed significant effect of sampling rate on performance (*p* < 0.001 for all combinations of Binary/Multiclass and sensor/smart watch). Though AUROC initially increased with sampling frequency, there seemed to be a plateau effect at higher sampling frequencies, with sampling rates above 10–20 Hz showing little to no improvement in average AUROC. Statistically significant differences from peak performance were observed for frequencies below 30 Hz (*p* ≤ 0.011) for Binary models and 20 Hz for Multiclass models (*p* ≤ 0.003). Tremor models based on data from the smart watch showed a similar trend, with statistically significant decreases occurring at sampling frequencies below 20 Hz (*p* ≤ 0.006).

**Fig. 3:**
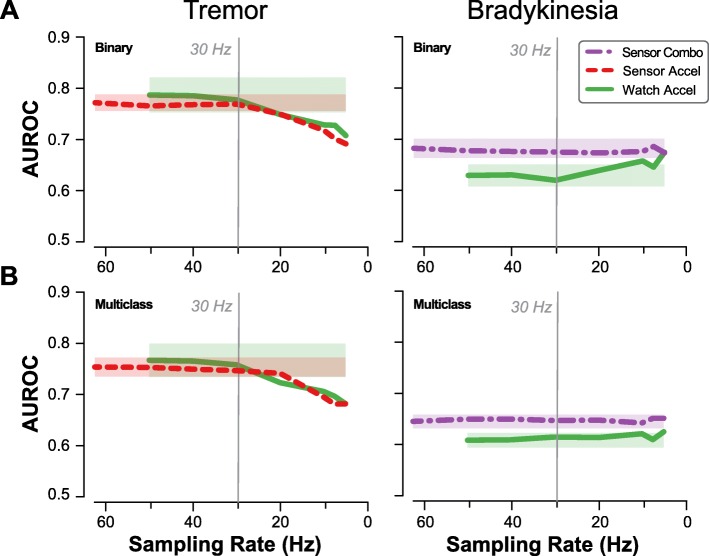
Effect of sampling rate. Model performance (AUROC) for (**a**) Binary and (**b**) Multiclass models of tremor and bradykinesia, using the previously determined sensor set for each symptom. Shaded regions depict a 95% confidence interval on the average AUROC centered at the original sampling rate. Decreasing sampling rate reduces ability to classify tremor beyond 20–30 Hz, with only slight impact on classifying bradykinesia. A 30-Hz sampling rate is sufficient to classify both symptoms using the BioStampRC sensor (Sensor) or smart watch (Watch)

For Binary models of bradykinesia, performance initially decreased then increased with lowering sampling rate. There was no significant effect of sampling rate on performance for bradykinesia models (*p* ≥ 0.48). Even sampling frequencies as low as 5 Hz resulted in no significant drop compared to peak performance (Fig. 3). Based on these results, the subsequent analysis of features sets was implemented on data downsampled to 30 Hz for all models.

### Feature analysis

Finally, we examined the trade-off between feature computation time and the performance of PD symptom models trained using those features. Feature categories were time-domain (Time), frequency-domain (Frequency), sample entropy (Entropy), cross-correlations (Correlation), signal derivative features (Derivative). Features were categorized this way to assess types of features, rather than the specific individual features selected here, and to group features requiring similar pre-processing computations (e.g., applying a discrete Fourier transform to compute frequency-domain features). We wished to broadly assess the effect of increased computational cost of feature sets – represented by average computation time – on the performance of PD symptom models, while also providing some insight into the relative value of different feature types for training these models. For clarity, the results are presented only for models based on data from the skin-adhesive sensors.

**Fig. 4:**
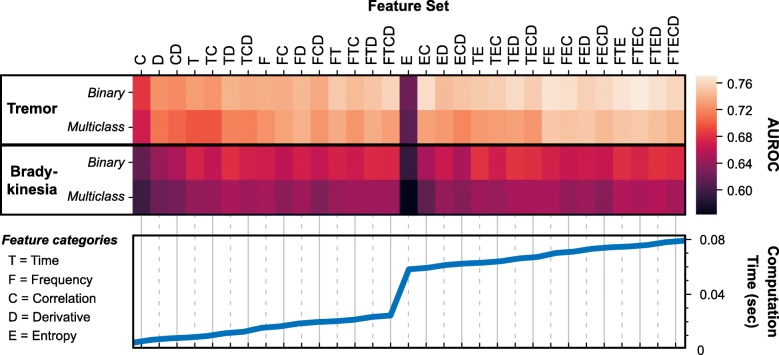
Feature set performance and computation time. Combinations of feature categories for the symptom models, ordered by total computation time. Model performance (AUROC) is computed for each model type and feature category combination. Generally, there is a trade-off between feature complexity and model performance, as more comprehensive feature sets improve AUROC but take longer to compute

Among the features sets, Correlation features had the lowest computation time, but also poor performance across all model categories. Entropy features had by far the largest computation time for any single feature set (54.9 ms) and the lowest performance when used alone, but led to potentially meaningful improvements in performance when combined with other feature types (Fig. 4).

To assess the extent to which feature computation time could be reduced before significantly impacting symptom detection, we performed statistical analyses on the effect of feature sets on model performance. Before these analyses, we removed any feature sets that showed a drop in performance for increasing computation time, since these would have no practical value when trying to reduce the computational cost of the features used.

For all four symptom/classification models, one-way rmANOVA identified a significant effect of feature set on model performance (*p* < 0.001). Peak performance for each model type was achieved with very similar feature sets - each peak set included Entropy and Time features, with Correlation and Frequency features often included as well (Table 6). Performance varied widely across feature sets for all models, with AUROC changing by at least 0.07 across feature sets for each model type. Most of this difference can be accounted for by the earliest added features. Near-peak performance (within 0.02 AUROC) could be obtained with sets of only one or two feature types and without the addition of computationally intense sample entropy features.

**Table 6:** Computation time and model performance for select feature sets

**TREMOR (Binary)**				**BRADYKINESIA (Binary)**			
**Features**	**Computation Time (ms)**	**AUROC**	**p**	**Features**	**Computation Time (ms)**	**AUROC**	**p**
C	4.41	0.68	< 0.001*	C	4.41	0.61	0.001*
D	6.47	0.73	< 0.001*	D	6.47	0.64	0.006*
T	8.15	0.73	0.001*	CD	7.55	0.65	0.005*
DT	11.29	0.74	0.018†	T	8.15	0.67	0.099
DF	18.42	0.75	0.022†	DT	11.29	0.68	0.174
FT	20.09	0.75	0.004*	ET	63.02	0.68	0.222
CDFT	24.31	0.76	0.040†	*DET*	*66.17*	*0.68*	*–*
CE	59.29	0.77	0.126				
EF	70.15	0.77	0.155				
EFT	74.98	0.77	0.071				
*CEFT*	*76.05*	*0.77*	**–**				
**TREMOR (Multiclass)**				**BRADYKINESIA (Multiclass)**			
**Features**	**Computation Time (ms)**	**AUROC**	**p**	**Features**	**Computation Time (ms)**	**AUROC**	**p**
C	4.41	0.67	0.001*	C	4.41	0.59	< 0.001*
D	6.47	0.71	< 0.001*	D	6.47	0.62	0.010*
DT	11.29	0.72	0.001*	CD	7.55	0.62	0.001*
F	15.28	0.73	0.014†	T	8.15	0.64	0.011*
CF	16.35	0.73	0.015†	DT	11.29	0.65	0.152
DF	18.42	0.74	0.037†	FTE	74.98	0.65	0.189
EF	70.16	0.75	0.234	*DEFT*	*78.11*	*0.66*	–
DEF	73.30	0.75	0.226				
*CEFT*	*76.05*	*0.75*	–				

Total computation time and average AUROC for each combination of feature categories (includes tri-axial and magnitude features). Only features combinations showing improved performance with increasing computation time were included. T = Time, F = Frequency, C = Correlation, D = Derivative, E = Entropy. Asterisk (*) indicates significant difference after Holm-Bonferroni correction (α = 0.05) from the best performing feature set, marked in italic. Dagger (†) indicates additional significant differences when not controlling the family-wise error rate

**Fig. 5:**
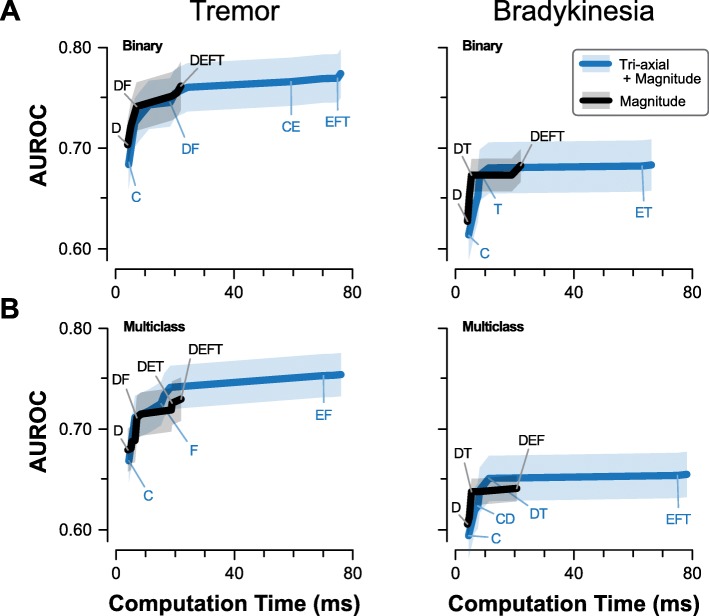
Effect of magnitude-only features. Relationship between feature computation time and model performance (AUROC) for (**a**) Binary and (**b**) Multiclass models of tremor and bradykinesia. Shaded regions depict a 95% confidence interval of the mean AUROC. Features derived from the signal magnitude alone can achieve similar performance to combined tri-axial and magnitude features at a fraction of the computation time

Using only features based on signal magnitude could be a method to provide greater richness of features with reduced computational cost. When features were computed from signal magnitude only (for applicable feature categories), the resulting models generally met or exceeded performance of full feature sets with similar computation times. The peak performance of magnitude-based feature sets was lower than the peak performance among full feature sets for each model category (Fig. 5), with the differences in peak performance more pronounced for Multiclass models. However, none of these differences were statistically significant (*p* ≥ 0.11) and represented at most a change of 0.02 in the mean AUROC.

## Discussion

Pairing novel wearable sensors with machine learning algorithms is a promising approach to automated detection of PD symptoms, including tremor and bradykinesia. As monitoring strategies continue to develop, it is important to understand the trade-offs between simplified data collection strategies and model accuracy. Our goal was to simplify the data measurement characteristics within our dataset by assessing the effects of sensor type and the sampling rate (which drain battery and data storage of the sensors), as well as the number and type of features (which are costly to compute and may lead to overfitting).

First, we found that the effect of sensor types depends on the symptom that is being monitored. For detecting tremor, models trained on data from an accelerometer only – whether from a smart watch on the wrist or a flexible sensor on the hand – performed comparably to models trained using combined data from an accelerometer and gyroscope. Conversely, symptoms of bradykinesia were better detected using both sensors (combined accelerometer-gyroscope data) than accelerometer data alone. A possible explanation is in the way symptoms of tremor and bradykinesia are defined. PD tremors have characteristic frequency and severity scores of tremor are defined in the MDS-UPDRS based on certain amplitudes. These fluctuations are captured by both gyroscope and accelerometer sensors and seem to be detected similarly when using either sensor type alone. Bradykinesia is defined as slowness to voluntarily initiate movement, with decreasing speed and amplitude over time [23, 24]. In addition, bradykinesia-focused items in the MDS-UPDRS generally have more subjective descriptions of different scores than tremor-focused items. Since the description and scoring of bradykinesia is dependent on the intended movement, richer movement data may be necessary to improve detection and scoring of bradykinesia. Angular velocity signals from gyroscope may also better capture changes in speed and amplitude characteristic of bradykinesia. These results align with previous studies finding high correlation between gyroscope features and clinical scores of bradykinesia [25, 26].

Second, we found that a minimal sampling rate of 30 Hz was needed to classify the presence and severity of tremor, whereas altering sampling rate between 5 and 60 Hz did not significantly impact ability to classify bradykinesia. Bradykinesia models need to identify low-frequency and low-amplitude movements, while tremor models must distinguish relatively high-frequency movements from non-tremor movements, which mirrors our findings. Typical human movements have peak frequencies in the range of 0–5 Hz [27], and would be captured reasonably well across the sampling rates tested here. Previous studies have noted that characteristic PD tremor frequency is 4–6 Hz [28, 29], but may have additional frequency components in the range 1–30 Hz [29]. According to the Nyquist sampling theorem [30], the 30 Hz sampling rate determined here is only sufficient to analyze signal frequencies up to half that rate, or 15 Hz. An ideal sampling rate captures the characteristic frequencies of tremor as well as just enough additional information to separate tremor from other movements with similar peak frequencies. Higher frequency components may not have been necessary to characterize the tremors and other movements of participants in this study.

Third, we considered the application of real-time symptom monitoring, where features are computed by the wearable device system and computational cost must considered. Real-time monitoring of symptoms could be useful for providing feedback to patients (such as helping them recognize activities that prompt symptoms) or for prompting patient feedback to improve the model (such as verifying that model predictions are correct or incorrect) [31]. Appropriate choices of features and models help to maintain accuracy while minimizing power drain on the device and resulting instances of missing data [15, 31, 32]. In the current study, we examined the effect of feature complexity (as measured by computation time) on classification of symptoms. Computation time varied among features, with entropy features taking substantially longer to compute. We found that models monitoring tremor symptoms showed significantly decreased performance once entropy features were removed, while significant performance drops were not observed for bradykinesia models until reaching lower computation times. For tremor detection, complex features such as entropy can boost accuracy as long as computational cost is not a limiting factor. In contrast, bradykinesia was sufficiently estimated by simpler features capturing the amplitude and variability of movements. The features considered here were certainly not exhaustive – new, novel types of features may be crucial to improve the observed AUROC values. Nor did we attempt to select the individual features for each model type that would maximize the AUROC. Future work may consider pairing feature selection techniques with an analysis of computational costs.

Across analyses we found comparable performance of models using only accelerometer data, whether obtained from the BioStampRC or the smart watch device. Though these devices were placed in slightly different locations (dorsal hand vs. wrist) and worn in different ways (adhered to the skin vs. wristband), these factors did not impact our conclusions. The changes in model performance we observed are therefore likely to be generally applicable to wearable sensors worn at the distal upper extremity and not only specific to the exact devices used in this study.

We chose to use AUROC as a performance metric because it captures model performance across all possible decision thresholds. Though a balance between sensitivity and specificity is generally preferable, different thresholds may be useful in certain clinical contexts (e.g. favoring sensitivity over specificity for screening tests). The actual impact of differences in AUROC will be highly dependent on the clinical context. However, performance of 0.7–0.8 is often considered “acceptable” while performance greater than 0.8 is considered “excellent” [33]. For our purposes here, we qualitatively consider a change of 0.05 in the AUROC to be potentially meaningful in a clinical context. By that criterion, most of our models showed little to no meaningful impact of simplifying the data collection parameters – except after fairly substantial reductions in sampling rate and number of feature types. We refrain from making specific recommendations about the minimum data collection parameters for PD symptom detection, since different modeling approaches and data sets are likely to yield higher or lower performance metrics and different sensitivities to these parameters. However, our general finding of diminishing returns to expanded data collection parameters when using wearable devices to monitor PD symptoms suggests that the cost of using additional hardware, higher sampling rates or numerous real-time feature computations should be carefully balanced against the potential impact on recording duration and participant adherence.

Though previous studies have explored the feasibility of wearable data for symptom monitoring, much less attention has been given to identifying the relative contributions of various settings and data to model performance. An exception is in examining the number and placement of devices for detecting symptoms. Previous studies have demonstrated that relative utility of data is highly dependent on body location and the intended application [22, 34]. For instance, sensors collecting data from a distal limb location are most effective for identifying symptoms in that limb [24], whereas more proximal sensor locations may be helpful for monitoring gait and posture [35]. This study focused on motor symptoms of the upper limb using a flexible sensor on the hand, but other sensor placements may be relevant for monitoring global presentations of symptoms. Incorporating additional types of sensors may improve detection accuracy, such as utilizing EMG data in tremor classification [36]. Note that the optimal data collection strategy may vary for other symptoms of PD (e.g., freezing of gait, postural instability, muscle rigidity, dystonia).

We believe that our strategy for simplifying data measurement characteristics can be applied broadly for different applications and performance requirements. However, the minimal characteristics may differ with the intended use of the system technology. Applications demanding higher diagnostic accuracy could benefit from increased measurement resolution. Furthermore, while we evaluated changes via AUROC statistics, this methodology can also be implemented for alternative metrics of model performance (e.g. F1-score, positive predictive value, etc.). We encourage investigators to use metrics that are most appropriate for their studies. Future work will determine whether the specific results presented here are replicated for other metrics.

The level at which computational burden becomes prohibitive can vary depending on the symptom and mode of operation. In one scenario, a clinician performing infrequent laboratory assessments has access to offline computational processing and may not be concerned about battery depletion or wired sensors during controlled, short-duration tasks. In another scenario, a patient wearing a continuous monitoring device in the community would benefit from real-time tracking of their symptoms, enabling them to have more data-driven discussions with their physician regarding experienced PD symptoms and their medication regimen. To achieve the second case, careful consideration of both the data measurement characteristics and the accuracy of the results is necessary. Here we have presented an initial analysis of how sensor type, sampling rate, and feature complexity might be taken into account, depending on the monitoring target. Future studies using larger amounts of data suitable for deep learning approaches may demonstrate additional benefit to high-resolution data or feasibility of lower-resolution approaches.

### Limitations

A primary limitation of this study is the small sample size. More data for training would likely improve the accuracy of the machine learning models to classify the presence and severity of tremor or bradykinesia. However, because we examined the relative contributions of data measurement characteristics to symptom detection accuracy for individuals with varying symptom severity, we expect the general trends to hold for larger training sets. However, future work may consider larger, separate training sets to determine data collection parameters for different levels of symptom severity. This would likely improve the resolution and accuracy for estimating the presence and severity of a targeted motor symptom.

Across these analyses it is important to note that the lack of a statistically significant difference between two model training datasets does not imply equivalence between the two models. Generally, when we did not find performance differences between a pair of models to be statistically significant, the magnitude of those differences was also small (< 0.02 AUROC). However, this does not mean that data with those measurement characteristics are equivalent in all possible modeling contexts. The basic trends in model performance across changing data measurement characteristics are still likely to apply more widely.

Another limitation is that training data for the machine learning model was collected during a standardized motor assessment, and patients were performing selected tasks in a supervised, clinical environment. This is relevant when considering a system for real-world monitoring of PD. We have previously shown that models trained on activities performed in a lab do not always generalize to activities performed at home [37]. Though the study tasks were designed to approximate naturalistic behavior, it is still critical to validate the performance of the any symptom detection model during day-to-day activities in the community. Indeed, a recent review of wearable technology to detect bradykinesia and rigidity found that very few studies investigate symptom detection in unsupervised, free-living settings [38]. Future work will examine in greater detail methods to adapt models trained on in-clinic data collected during specific tasks to symptom monitoring during free-living behaviors.

## Conclusions

To facilitate continuous, real-world monitoring of PD symptoms, wearable sensors should be unobtrusive and able to record the movement data for days and up to weeks without need for removal and replacement. Data measurement characteristics can be selectively reduced without significantly impacting model performance. This approach demonstrates a crucial step to improve power consumption and memory usage for new classes of remote health monitoring and automated diagnostic systems.

## Data Availability

The dataset used to support the findings of this publication are available from the Michael J. Fox Foundation but restrictions apply to the availability of these data, which were used under license for this study. The Michael J. Fox Foundation plans to release the dataset used in this publication alongside a significant, additional portion of related PD data from a separate smart watch analysis as part of a community analysis in the larger CIS-PD study timeline. However, data are available from the authors upon reasonable request and with permission from the Michael J. Fox Foundation.

## Abbreviations

AUROC: Area Under the Receiver Operator Characteristic curve
Accel: Accelerometer data condition
Combo: Combined accelerometer and gyroscope data condition
Gyro: Gyroscope data condition
MDS-UPDRS: Movement Disorder Society – Modified Parkinson’s Disease Rating Scale
PD: Parkinson’s disease
RF: Random Forest
rmANOVA: repeated measures Analysis of Variance
Watch: Smart watch data condition (accelerometer)
